# Case for improving respectful care: results from a cross-sectional survey of person-centred maternity care in rural South Africa

**DOI:** 10.1136/bmjph-2024-001086

**Published:** 2024-08-24

**Authors:** Tanya Doherty, Christiane Horwood, Sphindile Mapumulo, Lyn Haskins, Samuel Manda, Loveday Penn-Kekana, Silondile Luthuli, Veronique Filippi

**Affiliations:** 1 Health Systems Research Unit, South African Medical Research Council, Tygerberg, South Africa; 2 Paediatrics and Child Health, University of Cape Town, Cape Town, South Africa; 3 School of Public Health, University of the Western Cape, Cape Town, South Africa; 4 Centre for Rural Health, University of KwaZulu-Natal, Durban, South Africa; 5 Statistics Department, University of Pretoria, Pretoria, South Africa; 6 London School of Hygiene and Tropical Medicine, London, UK

**Keywords:** Public Health, Cross-Sectional Studies, Public Health Practice

## Abstract

**Introduction:**

Despite a supportive policy environment, little attention has been paid to how to operationalise respectful maternity care in South Africa. This research provides a quantitative, baseline measure of women’s perceptions of person-centred maternity care (PCMC) to identify areas of focus for a participatory intervention with maternity teams.

**Methods:**

A facility-based cross-sectional survey of postpartum women within 9 weeks postdelivery in two rural districts of KwaZulu-Natal. 908 postpartum women were recruited from postnatal clinics and neonatal units in the district hospitals. The primary outcome was PCMC measured on 30 items with a 4-point Likert scale (0–3). Mixed-effects linear regression analysis was used to identify predictor variables that were significantly associated with PCMC.

**Results:**

The mean PCMC score was 55.5 (SD 13.6) out of 90 and was significantly higher for women who had caesarean deliveries compared with vaginal births (58.0 (SD 12.8) and 54.5 (SD 13.7), respectively). Around one-fifth of women reported verbal abuse at least once. Over half of women felt that their health information would be kept confidential all of the time, less than 15% of women were allowed to have a companion with them during labour or delivery and less than half of women felt they could completely trust their providers all of the time. Attending eight or more antenatal visits, having a caesarean delivery, being in the age group 30–45 and being in the middle wealth quintile were independently associated with a higher PCMC score while delivering at night was associated with a lower score.

**Conclusion:**

Women attending rural health facilities experience disrespect and lack of trust in an environment where they have little involvement in decisions about their care and feel unable to ask questions of their providers. These findings support the need for interventions addressing organisational cultures that allow disrespect within maternity units.

**WHAT IS ALREADY KNOWN ON THIS TOPIC**

**WHAT THIS STUDY ADDS**

- Women attending rural health facilities experienced disrespect, lack of support and trust in their providers.
- Experiences of care in childbirth were worse for women who had vaginal deliveries compared with women who had caesarean deliveries and for women who delivered during the night.
- Less than 15% of women were allowed to have a companion with them during labour or delivery despite the guidelines recommending this practice.
- Women have little involvement in decisions about their care and feel unable to ask questions of their providers.

**HOW THIS STUDY MIGHT AFFECT RESEARCH, PRACTICE OR POLICY**

- These findings support the need for interventions addressing organisational cultures that allow disrespect within maternity units.
- Particular aspects of care that require attention include fostering trust with providers and respectful communication, particularly for women having a vaginal delivery, as well as openness to companionship in labour and delivery.
- Interventions to improve respectful care should include maternity staff working night shifts.

## Introduction

 At the global level, almost all women (88%) are now receiving at least one antenatal visit and a substantial majority (78%) deliver in health facilities.^1^ Attention has shifted from an emphasis on increasing the utilisation of maternity services to a more nuanced focus on enhancing the quality of care provided. This shift has been accompanied by growing evidence of the poor treatment of women during facility-based childbirth.^2^,^4^ There have also been progressive changes to terminology from more negative terms such as obstetric violence and mistreatment^5^ to more positive framing of respectful and person-centred maternity care (PCMC).^6^ The WHO intrapartum care guideline of 2018 recommends respectful maternity care (RMC) for all women, which is care that maintains ‘dignity, privacy and confidentiality, ensures freedom from harm and mistreatment and enables informed choice and continuous support during labour and childbirth’.^7^

Disrespect and abuse in maternity services in South Africa (SA) has been well described over several decades and while not unique to SA, research has found that it is deeply rooted in the country’s complex sociopolitical landscape.^8^ An unequal healthcare system, inherited from the apartheid era, is characterised by maldistribution of health workers and resources between the public and private sector.^9^ Much of the research on the quality of maternity care in SA has used qualitative research designs and focused on urban-based midwife obstetric units (MOUs), a model where midwives provide care for low-risk women in stand-alone maternity units. One of the first post apartheid research studies (published in the late 1990s) documenting women’s experiences of maternity care described nurses’ verbal and physical violence towards women as a means of creating social distance and maintaining a relationship of power.^8^ Other neglectful and abusive practices commonly reported by mothers in SA include unfriendly provider attitudes, poor communication, failure to inform women about their care and lack of privacy but may also include physical and verbal abuse.^10 11^ The majority of nurses and midwives in SA are women living in environments where they may themselves be experiencing gender-based violence.^8 12^ A recent qualitative study conducted in MOUs in Cape Town described patient neglect as a form of normalised violence within maternity settings.^13^

Three decades of research on poor quality of maternity care in SA and increased global attention on the importance of the experience of care as a component of quality maternity care^7^ have led to revisions in national guidelines and policies to specifically include a focus on respectful care. The SA maternal, perinatal and neonatal health policy, updated in 2021, includes a specific policy statement on RMC.^14^ Similarly the fourth edition of the guidelines for maternity care in SA published in 2016 states that ‘Health workers administering care to pregnant women must demonstrate respect and a genuine interest in their clients and avoid an arrogant, rude or judgemental attitude. This applies even in the context of a poor working environment or perceived unsafe practices of certain pregnant women’.’^15^

An additional factor influencing the policy shifts towards improved experience of care is the high burden of medicolegal claims in the public sector, the majority of which are obstetric related and amounted to R77 billion (approximately US$4 billion) in 2023. Disrespectful care and poor clinical care are closely linked, for example, when mothers are left unattended for long periods and feel unable to voice their concerns, this leads directly to poor outcomes for mothers and newborns.^16^ In addition, women and families who have not received explanations about an adverse outcome may be more likely to litigate. Implementing the South African patients’ rights charter, which states that patients have the right to care by healthcare workers ‘that demonstrates courtesy, human dignity, patience, empathy and tolerance’’^17^ is one of the proposed solutions to the obstetric medicolegal crisis.^18^

There has been very little quantitative measurement of women’s experiences of care during childbirth in SA and a lack of focus on rural contexts where challenges to the provision of quality maternity care are even greater than urban areas.^19^ Furthermore, in KwaZulu-Natal province, one in three women in the public sector (34.5%) has a caesarean delivery. This is the highest rate in the country with the national public sector rate for 2020 being 28%.^20^ Unlike most other countries in sub-Saharan Africa, there is good access to caesarean delivery in SA with 97% of births being attended by a skilled birth attendant.^21^ However the caesarean delivery rate is far higher than the WHO-recommended rate of 10%–15% of all births^22^ and there are concerns about the safety of caesarean delivery in the public sector with the caesarean delivery case fatality ratio in 2021 being 204 deaths per 100 000 caesarean deliveries compared with a total maternal mortality ratio of 148 per 100 000 births in the same year.^20^ To date, there has been no research measuring women’s experiences of caesarean delivery in SA. This research aimed to provide a quantitative, baseline measure of PCMC in two rural districts in order to inform the future development of a participatory learning and action intervention to improve RMC.

## Methods

We undertook a cross-sectional survey using the PCMC tool developed by Afulani *et al*.^23^ This tool, and the derived PCMC scale, was developed in a rural and urban setting in Kenya^23^ and subsequently applied in India and Ghana.^24^ The development of the scale in Kenya followed standard procedures for scale development^25^ including psychometric analyses to assess the validity and reliability of the individual items. The final tool with a 30-item scale and 3 sub-cales correlated with global measures of satisfaction with maternity services.^23^

This survey was the first application of the PCMC tool in SA. It was undertaken between October 2022 and February 2023 to obtain a baseline measure of PCMC, as part of a before-and-after evaluation design for a facility-based participatory learning and action intervention with maternity unit staff.

### Study setting

The study was conducted in two rural districts of KwaZulu-Natal, SA (uMzinyathi and Zululand). These two districts were chosen based on high maternal and neonatal mortality levels, low population density, geographical remoteness and long travel distances between district-level and secondary-level referral hospitals. uMzinyathi is a deep rural and mountainous district with poor basic infrastructure, little economic growth and a low population density of 64 people per km^2^. 60% of the population lives below the lower poverty line.^26^

Zululand is the biggest district in Kwazulu-Natal making up 16% of the geographical area. It is a rural district with half of the area under the jurisdiction of traditional authorities. The population density is 60 people per square kilometre and 70% of the population live below the lower limit poverty line with two municipalities regarded as the poorest in the country.^27^

In total, there are nine district hospitals in the two districts where low-risk vaginal deliveries are conducted as well as low-risk elective and emergency caesarean deliveries. The delivery rooms within the district hospitals are well equipped and contain between 2 and 4 beds with curtains that can be pulled around the beds when a woman is in active labour.

All pregnant and lactating women and children under the age of 6 receive free healthcare in the public sector. Women with low-risk pregnancies receive antenatal care at primary care clinics and community health centres. District hospitals provide level 1 (generalist) services to inpatients and outpatients including obstetric care for women with low-risk pregnancies. District hospitals have between 30 and 200 beds, a 24-hour emergency service and an operating theatre. Generalist doctors (medical officers) provide the services together with advanced midwives, midwives, nurses and allied health professionals. There are no obstetric or anaesthetic specialists at the district hospital level. Most district hospitals also have community service doctors. These are doctors who have completed a 2-year internship postgraduation and are required to complete a further 1 year of community service.^28^

In 2016, 96% of all deliveries in the province took place in a health facility.^29^ Deliveries to adolescents and young women 10–19 years were 22% and 20% of total in-facility deliveries in Zululand and uMzinyathi, respectively, in 2020.^30^ The number of public sector medical practitioners per uninsured population is among the lowest in the country at 14.8 per 100 000 for Zululand and 17.6 per 100 000 for uMzinyathi in 2020 compared with a national average of 33.6.^31^

According to the latest Saving Mother’s Report, the institutional maternal mortality ratio for KwaZulu-Natal province for 2020 was 116 and the most common cause of death was non-pregnancy-related infections. The HIV prevalence among pregnant women in 2022 was 38% in Zululand and 31% in uMzinyathi.^32^ The caesarean delivery rate for KwaZulu-Natal in 2020 was 35% and the caesarean delivery case fatality rate was 123 per 100 000 caesarean deliveries.^20^

### Sample size

The sample size for this baseline PCMC survey was determined based on 80% power and a significance level of 0.05 to measure an expected baseline of 8.3 (out of 27) on the ‘communication and autonomy’’ dimension of the PCMC scale (measured as the sum of the nine items in this dimension, each on a 0–3 Likert scale) with an SD of 3.3. The expected baseline score was determined from the minimum country baseline from a multicountry application of the tool in Ghana, India and Kenya.^24^ Estimating a mean number of postnatal women per clinic of 100 over a 2-week data collection period (cluster size variation 50–120), controlling for the variable size of clusters (coefficient of variation for cluster size assumed at 0.5) and clustering (assumed intraclass correlation coefficient (ICC) of 0.08), required 680 women.

### Sampling and data collection

Women aged 16–49 years who had given birth in the previous 9 weeks were recruited from 37 postnatal clinics in the catchment areas of the district hospitals and the neonatal units in all 9 district hospitals. Consecutive women waiting for postnatal and immunisation services were approached until the required sample size was achieved. An initial screening question asked women where they had delivered and to be eligible to participate in the survey they must have delivered in one of the nine district hospitals. A total of 823 women were interviewed at postnatal clinics which do not perform deliveries. These women all delivered at one of the nine district hospitals. All women with newborns admitted to the neonatal units in the district hospitals during the period of data collection were invited to participate. A total of 85 women were interviewed in neonatal units within the same hospital where they had given birth. Recruitment at clinics and hospitals occurred over the same time period. A total of two women refused to participate in the survey.

The PCMC tool, consisting of the 30-item PCMC scale with additional demographic and quality of care questions relevant to SA, was administered on Android tablets with built-in range and logic restrictions. The tool was translated into IsiZulu and piloted in clinics to assess the understanding and accuracy of the translations. Trained field researchers administered the survey on tablets in IsiZulu in a private room within the health facilities or outside the facility if a private space was not available. The field researchers explained to women that they were not staff members of the health facility or department of health and would not report any survey responses back to the health facilities where women delivered. Women did not receive any compensation for participating. The final sample of women who participated in the survey was 908, larger than the required sample as we invited all women with newborns admitted to newborn units to participate.

### Primary outcome measure

The primary outcome measure was PCMC, measured on the 30-item PCMC scale. Each item has a 4-point response scale—ie, 0 (‘no, never’), 1 (‘yes, a few times’), 2 (‘yes, most of the time’) and 3 (‘yes, all the time’). The scale has three domains: dignity and respect, communication and autonomy and supportive care. The overall PCMC score was taken as a summative score from the responses to individual items in the 30-item PCMC scale (with negative items reverse coded—ie, questions that were framed negatively, such as waiting time, had to be recoded so that high numbers represent good care). There are items that have a ‘not applicable’ response option. We included these items because they are relevant to PCMC even if they did not apply to all respondents. As recommended by the scale developers these ‘not applicable’’ options were recoded into the upper middle category (ie, yes, most of the time).^23^

The minimum possible score on the PCMC scale is 0 and the maximum possible is 90, with a low score indicating poor PCMC.

### Measurement of independent predictors

We examined potential predictors of PCMC including demographic variables such as age, parity and marital status and measures of socioeconomic status (ie, education, employment and household wealth). We also captured variables measuring antenatal care attendance, delivery, facility and provider characteristics. Delivery characteristics included mode of delivery (vaginal or caesarean) and whether the birth took place during the day (7:00–19:00 hours) or during the night. Provider characteristics were health worker type (nurse/midwife or doctor) and gender. We present demographic characteristics and the distribution of individual scale items by mode of delivery to highlight differences in experiences of care between women who had vaginal and caesarean deliveries.

### Statistical analysis

We followed the approach used by the Demographic and Health Surveys Programme^33^ to construct a wealth index. We used the first component of a principal component analysis statistical procedure to construct a wealth index for measuring the socioeconomic and living standards of households. We used the mother’s level of education and the household’s ownership of selected assets, such as refrigerator, vacuum cleaner, microwave oven, electric or gas stove, washing machine, watch, cellphone, bicycle, motorcycle/scooter and car or van; and household food security. Each of these was coded 1 if it was present, otherwise it was coded 0. We then subdivided the derived score into five equal parts to create wealth quintiles. We used Cronbach’s alpha to measure the level of internal consistency in the multiple Likert scale items in the PCMC scale and the domains.

Preliminary data analyses involved descriptive statistics and bivariate association between the PCMC scale or subscales and the potential predictors using two-sample t-tests and analysis of variance statistical techniques. A mixed-effects linear regression analysis with random effects for health facilities to account for possible clustering of individual women’s responses in each hospital was used to examine associations between PCMC scores and possible predictor variables.

## Results

Table 1 shows the demographic characteristics of respondents by mode of delivery. The mean age was approximately 26 years with 15% of the sample being young women (16–19 years) and the average parity was two children. Less than a quarter of women were married or living with a partner and approximately half of the women had completed secondary school education. There was no significant difference between any demographic characteristics by mode of delivery. Women who had a caesarean delivery were more likely to have a delivery conducted by a male provider, by a doctor and during daytime hours, compared with women who had vaginal deliveries.

**Table 1:** Demographic characteristics of respondents and potential predictors of PCMC

	Vaginal delivery (n=662)	Caesarean delivery (n=246)
Total sample	n (%)	n (%)
Age		
Mean (SD)	26.5 (6.5)	26.8 (6.9)
16–19 years	103 (15.6)	37 (15.0)
20–29 years	349 (52.7)	122 (49.6)
30–45 years	210 (31.7)	87 (35.4)
Parity, n (mean (SD))	2.3 (1.3)	2.1 (1.2)
Marital status		
Living with a partner or married	123 (18.6)	57 (23.2)
Single or not living with a partner	539 (81.4)	189 (76.8)
Education		
Never attended school or primary	28 (4.2)	5 (2.0)
Some secondary school	278 (41.9)	92 (37.4)
Completed secondary school	319 (48.2)	132 (53.7)
Tertiary education	37 (5.6)	17 (6.9)
Any paid work in the previous 12 months		
Yes	173 (26.1)	52 (21.1)
No	489 (73.9)	194 (78.9)
Wealth quintile		
Poorest	153 (23.1)	42 (17.1)
Poor	124 (18.7)	45 (18.3)
Middle	126 (19.0)	55 (22.4)
Rich	135 (20.4)	51 (20.7)
Richest	124 (18.7)	53 (21.5)
Number of antenatal care visits		
No antenatal care	5 (0.8)	0
Less than 8 visits	370 (55.9)	130 (52.9)
8 or more visits	286 (43.3)	116 (47.1)
District		
uMzinyathi	332 (50.1)	113 (45.9)
Zululand	330 (49.9)	133 (54.1)
Delivery provider		
Midwife/nurse	615 (94.0)	4 (1.6)*
Doctor	39 (5.9)	242 (98.4)
Delivery provider gender		
Male	118 (17.8)	184 (76.7)
Female	544 (82.2)	56 (23.3)
Timing of delivery		
Daytime (7:00–19:00 hours)	368 (55.6)	159 (64.6)
Night-time (19:00–7:00 hours)	294 (44.4)	87 (35.4)

* Midwives do not perform caesarean deliveries in South Africa so these responses reflect that women may have thought that a female doctor was a midwife.

PCMCperson-centred maternity care

Online supplemental table 1 shows the distribution of all the PCMC variables by mode of delivery. Just over two-thirds of women in both groups felt that they were treated with respect all the time. There were several areas where PCMC was poor irrespective of mode of delivery. Around one-fifth of women reported verbal abuse at least once and 7% reported any physical abuse. A little over half of women felt that their health information would be kept confidential all of the time, less than 15% of women were allowed to have a companion with them during labour or delivery and less than half of women felt that they could completely trust their providers all of the time. Less than half of women reported that there was water at the facility all of the time. Perceptions of overcrowding were common with 55% of women who had vaginal births and 65% of women who had caesarean deliveries reporting that the labour and postnatal wards were crowded.

Differences in experiences of care were noted for several items between women who had vaginal births and those who had caesarean deliveries. Women who had caesarean deliveries reported greater privacy during examinations in the labour room. Just over half of women who had vaginal deliveries and a quarter of women who had caesarean deliveries reported that providers never introduced themselves. 86% of women who had vaginal births did not feel they could be in a position of their choice during delivery, and around one-third of women who had a vaginal delivery and half of women who had caesarean deliveries felt that providers always involved them in decisions about their care. Almost half of women who had vaginal births and a third of women who had caesarean deliveries said providers never asked permission before doing examinations or procedures on them. Similarly, almost half of women who had vaginal births and one-third of women who had caesarean deliveries felt they could not ask the doctors or nurses any questions. 28% of women who had vaginal deliveries and 10% of women who had caesarean deliveries felt providers did not do their best to control their pain.

Two additional questions were added to the survey for this rural SA sample related to whether women shared a bed and whether women would choose to give birth in the same facility again. Roughly 1 in 10 women reported that they had shared a bed with another woman at some time during their stay in the hospital. One in five women said if they had a choice they would definitely or probably choose not to give birth in the same facility again.

Mean PCMC scores on the full scale and subscales are shown in table 2 for all women and by mode of delivery. The mean total PCMC score was significantly higher for women who had caesarean deliveries compared with vaginal births (58.0 and 54.5, respectively). The mean score for the communication and autonomy subscale was also significantly higher among women who had caesarean deliveries. For the other two subscales, the average scores were also higher among women who had caesarean deliveries but the differences were not statistically significant.

**Table 2:** Internal consistency values and the distribution of full PCMC scale and subscales

	Cronbach’s α value	Mean raw scores (SD; min–max range)
Full sample (n=908)		
Full PCMC scale*	0.86	55.5 (13.6; 16–85)
Dignity and respect†	0.66	15.0 (3.1; 0–18)
Communication and autonomy‡	0.71	14.5 (5.5; 2–27)
Supportive care§	0.79	25.9 (7.5; 6–45)
Vaginal deliveries (n=662)		
Full PCMC scale*	0.86	54.5 (13.7; 16–85)
Dignity and respect†	0.66	14.9 (3.3; 0–18)
Communication and autonomy‡	0.71	13.8 (5.5; 2–27)
Supportive care§	0.79	25.8 (7.7; 7–45)
Caesarean deliveries (n=246)		
Full PCMC scale*	0.86	58.0 (12.8; 26–84)
Dignity and respect†	0.63	15.3 (2.8; 4–18)
Communication and autonomy‡	0.68	16.4 (5.2; 4–26)
Supportive care§	0.79	26.3 (7.2; 6–42)

* Full PCMC scale has 30 items, each on a scale of 0–3; therefore, the scores range from 0 to 90.

† The dignity and respect subscale has 6six items and scores range from 0 to 18.

‡ The communication and autonomy subscale has 9nine items and scores range from 0 to 27.

§ The supportive-care subscale has 15 items and scores range from 0 to 45.

PCMCperson-centred maternity care

**Figure 1:**
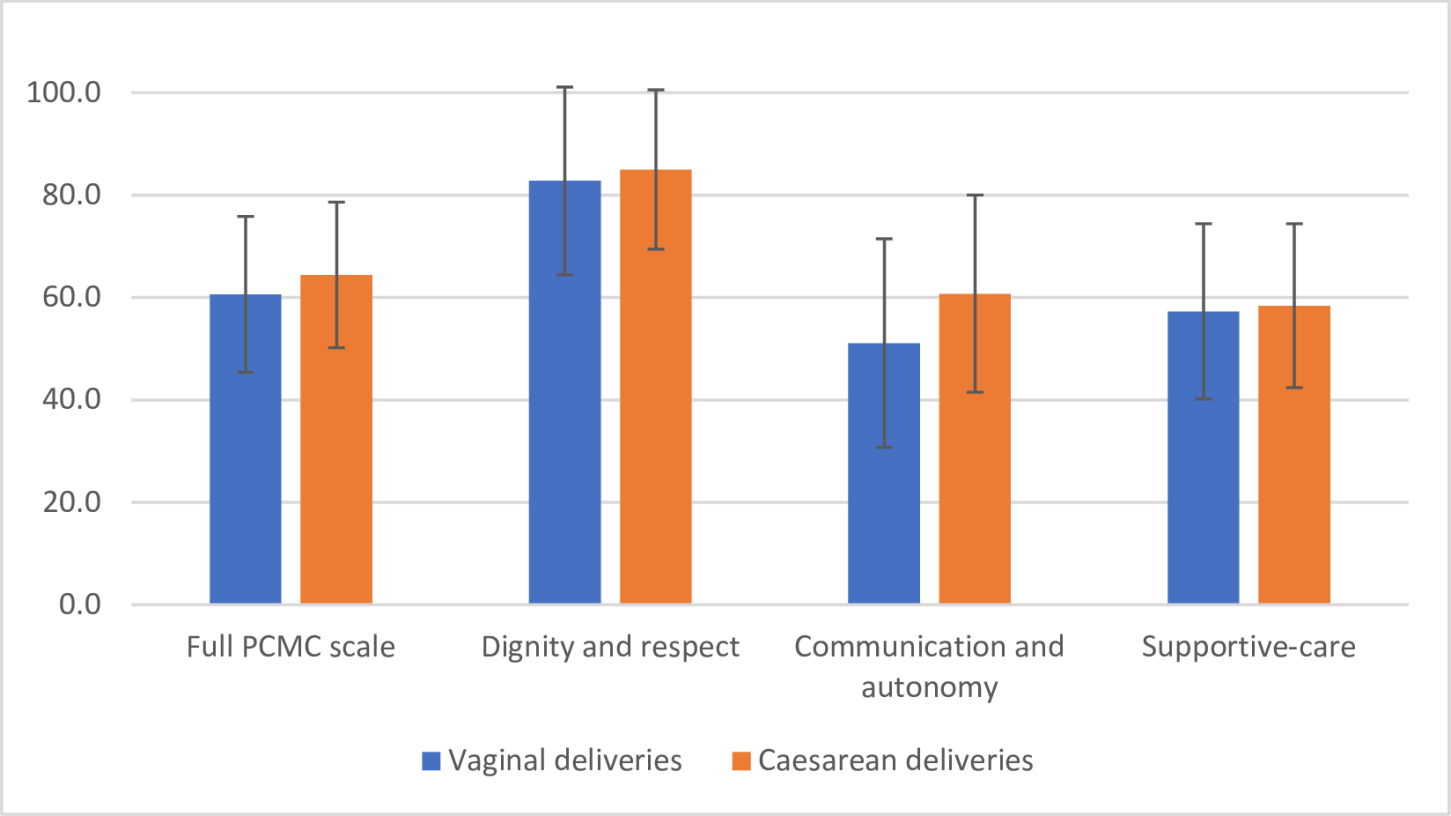
Rescaled scores on person-centred maternity care (PCMC) full scale and subscales. Rescaled scores are calculated as the fraction of the total possible score on each domain and normalised to 100. Error bars are rescaled SD values.

To enable comparison across the domains, scores were rescaled (ie, shown as a fraction of the total possible score on that domain and normalised to 100) and shown in figure 1. The figure shows that the highest scores were obtained for the dignity and respect subscale at over 80% of the maximum possible score. The lowest performing area was communication and autonomy which was only 50% of the maximum possible score for women who had vaginal deliveries.

Table 3 shows the mean PCMC score by selected predictors. Women aged 30–45 years had a higher mean PCMC score compared with women aged 16–19 years. Women in the middle, rich or richest quintiles had a higher mean PCMC score compared with the poorest women. Women who had eight or more antenatal visits, caesarean delivery, where the delivery provider was a doctor and those who delivered during the daytime had a higher mean PCMC score compared with women with less than eight antenatal visits, who had a vaginal delivery, where the delivery provider was a nurse or midwife and those who delivered during the night.

**Table 3:** Distribution of person-centred maternity care (PCMC) scores by key predictors

	Mean PCMC score out of a maximum of 90 (SD)	Significance level(p value)
Total sample (n)	908	
Age		
16–19 years	53.1 (12.8)	Ref
20–29 years	54.9 (13.6)	0.175
30–45 years	57.5 (13.6)	0.002
Parity		
1	54.5 (12.9)	Ref
2	55.3 (13.8)	0.485
3	56.9 (14.2)	0.054
4+	56.5 (13.7)	0.152
Marital status		
Living with a partner or married	57.1 (14.4)	Ref
Single or not living with a partner	55.1 (13.4)	0.073
Education		
Never attended school or primary	52.7 (13.9)	Ref
Some secondary school	55.8 (13.3)	0.211
Completed secondary school	55.2 (13.5)	0.313
Tertiary education	57.2 (15.9)	0.136
Any paid work in the previous 12 months		
Yes	54.8 (13.9)	0.374
No	55.7 (13.5)	Ref
Wealth quintile		
Poorest	52.6 (12.9)	Ref
Poor	55.3 (12.0)	0.064
Middle	57.1 (13.2)	0.001
Rich	55.4 (14.2)	0.045
Richest	57.2 (14.9)	0.001
Number of antenatal care visits		
Less than 8 visits	53.4 (13.2)	Ref
8 or more visits	58.1 (13.6)	0.000
Delivery type		
Vaginal delivery	54.5 (13.7)	Ref
Caesarean delivery	58.0 (12.8)	0.001
District		
uMzinyathi	54.7 (12.4)	Ref
Zululand	56.2 (14.6)	0.092
Delivery provider		
Midwife/nurse	54.6 (13.6)	Ref
Doctor	57.8 (13.2)	0.001
Delivery provider gender		
Male	56.5 (12.9)	Ref
Female	54.9 (13.9)	0.115
Timing of delivery		
Daytime (7:00–19:00 hours)	56.7 (13.6)	Ref
Night-time (19:00–7:00 hours)	53.8 (13.4)	0.002

Accounting for the effect of all other predictors, attending eight or more antenatal visits and being in the middle wealth quintile was independently associated with a higher score on the total PCMC and all three subscales. For the other covariates, differences were noted across the subscales (table 4).

**Table 4:** Results of the multivariate linear regression of PCMC and PCMC domains on selected covariates

	PCMC	Dignity and respect	Communication and autonomy	Supportive care
	Coefficient (95% CI)	Coefficient (95% CI)	Coefficient (95% CI)	Coefficient (95% CI)
Mode of delivery				
Vaginal delivery	Ref.	Ref.	Ref.	Ref.
Caesarean delivery	3.51*** (1.58 to 5.45)	0.35 (−0.11 to 0.80)	2.56*** (1.79 to 3.33)	0.64 (−0.43 to 1.72)
Antenatal care				
Less than 8 antenatal visits	Ref.	Ref.	Ref.	Ref.
8 or more antenatal visits	3.97*** (2.21 to 5.74)	0.87*** (0.46 to 1.29)	1.46*** (0.76 to 2.16)	1.60** (0.62 to 2.59)
Timing of delivery				
Daytime (7:00–19:00 hours)	Ref.	Ref.	Ref.	Ref.
Night-time (19:00–7:00 hours)	−2.02* (−3.74 to 0.29)	−0.12 (−0.53 to 0.28)	−0.90* (−1.59 to –0.22)	−1.00* (−1.96 to –0.04)
Age				
16–19 years	Ref.	Ref.	Ref.	Ref.
20–29 years	2.24 (−0.61 to 5.08)	0.49 (−0.18 to 1.17)	0.88 (−0.26 to 2.01)	0.85 (−0.73 to 2.43)
30–45 years	5.10** (1.33 to 8.87)	0.95* (0.06 to 1.85)	2.08** (0.58 to 3.58)	2.09 (−0.00 to 4.19)
Parity				
1	Ref.	Ref.	Ref.	Ref.
2	−0.49 (−2.87 to 1.88)	−0.37 (−0.94 to 0.19)	0.08 (−0.87 to 1.02)	−0.18 (−1.50 to 1.14)
3	−0.68 (−3.71 to 2.35)	−0.75* (−1.47 to 0.04)	−0.23 (−1.44 to 0.97)	0.29 (−1.39 to 1.98)
4+	−0.79 (−4.55 to 2.98)	−0.92* (−1.81 to 0.03)	0.16 (−1.33 to 1.66)	−0.03 (−2.13 to 2.06)
Marital status				
Living with a partner or married	Ref.	Ref.	Ref.	Ref.
Single or not living with a partner	−0.83 (−3.10 to 1.44)	−0.20 (−0.74 to 0.33)	−0.12 (−1.02 to 0.78)	−0.48 (−1.74 to 0.79)
Education				
No school or primary	Ref.	Ref.	Ref.	Ref.
Some secondary school	2.79 (−1.93 to 7.52)	−0.67 (−1.79 to 0.45)	1.49 (−0.39 to 3.36)	2.00 (−0.62 to 4.63)
Completed secondary school	0.58 (−4.28 to 5.44)	−0.66 (−1.81 to 0.49)	0.85 (−1.08 to 2.79)	0.44 (−2.26 to 3.14)
Tertiary education	3.18 (−2.82 to 9.17)	−0.99 (−2.42 to 0.42)	2.39* (0.01 to 4.77)	1.90 (−1.43 to 5.23)
Household wealth				
Poorest	Ref.	Ref.	Ref.	Ref.
Poor	2.16 (−0.60 to 4.92)	0.37 (−0.28 to 1.03)	0.38 (−0.72 to 1.48)	1.37 (−0.17 to 2.90)
Middle	3.82** (0.97 to 6.67)	0.74* (0.07 to 1.42)	1.44* (0.31 to 2.57)	1.61* (0.03 to 3.19)
Rich	1.84 (−1.05 to 4.73)	0.73* (0.05 to 1.41)	0.40 (−0.74 to 1.55)	0.64 (−0.97 to 2.25)
Richest	3.04 (−0.07 to 6.15)	0.52 (−0.21 to 1.25)	1.41* (0.18 to 2.65)	1.02 (0.72 to 2.76)
Constant	48.5*** (42.6 to 54.5)	14.8*** (13.4 to 16.1)	10.5*** (8.1 to 12.8)	23.4*** (19.9 to 26.9)
Observations	905	905	905	905

Adjusted for possible clustering of mother’s responses at the delivering health facility using a mixed effects model, treating a facility effect as random.

* p<0.05, **p<0.01, ***p<0.001.

PCMCperson-centred maternity care

Having a caesarean delivery and being in the age group 30–45 years was associated with a higher total PCMC score while delivering during the night was associated with a lower total PCMC score adjusting for the effect of all other predictors. For the dignity and respect subscale, being in the age group 30–45 years and being in the middle or rich wealth quintile was associated with a higher score on this subscale while having three or more children was associated with a worse experience of care. For the communication and autonomy subscale, a higher score was associated with caesarean delivery, being in the age group 30–45 years, having tertiary education and being in either the middle or richest wealth quintile. Delivering during the night was associated with a lower score for the communication and autonomy subscale. For the supportive care subscale, giving birth during the night was associated with a lower score (table 4).

## Discussion

Our results suggest that important areas for improvement exist within the South African maternity care context, in particular in relation to communication with women. Using a validated scale, we found a mean PCMC score of 55 (out of 90) with the domains of communication and autonomy and supportive care scoring lower than dignity and respect. The mean PCMC score for these rural districts in SA was lower than both the rural (59.5) and urban (60.2) samples in Kenya, higher than Ghana (46.5) and similar to India (55.8) where this tool has been previously applied.^24^

Areas of poor care that were common across all women irrespective of mode of delivery were fearing that their health information would not be kept confidential and having low levels of trust in their providers. Trust between women and providers has been described as a neglected yet important determinant of experiences of maternity care.^34^ We found that less than half of women reported that they could completely trust their provider all of the time. Lack of trust has major implications for women’s care-seeking and we found that one-fifth of our respondents would probably or definitely choose not to deliver at the same facility again if they had a choice which is severely limited in the public sector since it would require a long travel time to reach a different district hospital.

Women also reported a lack of basic infrastructure within health facilities such as a consistent supply of water and having to share a bed with another woman. The availability of water, sanitation and hygiene facilities in maternity units is essential for preventing infections. Our survey finding that less than half of women reported that there was water in the health facility all of the time they were there, is worse than the latest inspection report of the Office of Health Standards Compliance which found that 74% and 57% of health facilities in uMzinyathi and Zululand, respectively, were compliant with the required vital measures of maintenance of physical infrastructure and availability of back-up electricity and water supply.^35^ Research on obstetric disrespect and abuse has pointed to the stressors of under-resourced health systems in influencing health provider behaviours.^36^

The SA maternity care guidelines state that providers should ‘allow family and friends to provide companionship during labour’’,^15^ yet less than 15% of women reported being allowed to have a companion with them during labour or delivery. A labour companion can improve communication with health providers, articulate women’s concerns and provide practical help and support for women during labour. WHO recommends labour companionship as an intervention to improve respectful care, which has also been shown to improve maternal and perinatal outcomes.^37^ However, it is common for health workers to lack awareness about birth companions and express concern about overcrowding and privacy for other women in the labour ward, and this has hampered the implementation of birth companions in many settings,^38^ including in SA.

Experiences of physical abuse by women in our sample were relatively few (7%) which may indicate a shift from what has been reported from previous qualitative research in SA.^8^ However, verbal abuse remains common with around 20% of women, irrespective of the mode of delivery, reporting verbal abuse at least once. These rates are similar to what was found from the same survey tool applied in urban Kenya and India but higher than rates reported by women in rural Kenya and Ghana.^24^

Women who had caesarean births reported significantly better experiences of care compared with women who had vaginal births although these experiences were still far below acceptable quality of care. This is the first time experience of care among women who have had caesarean delivery has been measured in SA; however, a similar finding was reported from a study using the PCMC tool in Rural Kenya, Ghana and India^39^ and from a facility and community survey in Tanzania.^40^ The main areas where experiences of care were better for women who had caesarean deliveries were privacy during examinations, communication (providers introducing themselves, involving women in decisions, asking permission for examinations) feeling able to ask questions of providers and experiencing better control of pain. Women who had vaginal deliveries did not differ from women who had caesarean deliveries for any sociodemographic characteristics but there were important differences in type of health provider (doctor compared with midwives/nurses) provider gender (more males conducted caesarean deliveries) and timing of delivery (more caesarean deliveries occurring in the daytime). Provider interactions with women may differ when a complication or risk is identified which may partially explain the perception of better communication from women who had caesarean deliveries. Women who have caesarean deliveries have to go through a consent procedure with a health professional and may have experienced complications in labour and perceive the procedure to have been life-saving for themselves or their infants. Furthermore, they also stay longer in the hospital and therefore encounter a range of providers in labour and postnatal units all of which may influence their overall perception of communication and their sense of autonomy.

Besides the mode of delivery, we found several other factors independently associated with better experiences of care including older age, attending more antenatal visits, delivering during the daytime and a higher socioeconomic status. A cross-sectional facility-based survey in Ethiopia also reported better experiences of RMC among women who delivered during the daytime.^41^ Night shifts typically have less management supervision and providers may suffer from exhaustion particularly if they have household and family responsibilities that prevent them from resting during the day when they are off shift. In SA, studies have found a high level of personal and work-related burnout and stress among health workers,^42^,^44^ which may provide some context for the lack of empathy displayed by some health workers.

Our findings complement previously conducted qualitative research in SA describing women’s experiences of care in childbirth.^8 10 13^ While the qualitative research has described both verbal and physical abuse, our survey found that reports of physical and verbal abuse were relatively uncommon but the different methodologies used prevents us from concluding whether this represents an improvement.^45^

The findings from this baseline survey point to important gaps that will be the focus of the next phase of this research, which aims to develop a participatory intervention to improve RMC within rural maternity teams. Despite the well-documented manifestations and structural drivers of mistreatment and a supportive policy environment in SA, very little attention has been paid to how to operationalise RMC^46^ among multidisciplinary teams of health workers in public sector maternity units; specifically how to change organisational cultures that normalise disrespect and abuse.^36^ Previous research in SA has focused on promoting birth companions through an educational package provided to maternity staff but found no impact of the intervention after 8 months. Challenges identified included the lack of team consensus to implement change, high staff turnover and lack of support from hospital management.^47^ The existing global evidence synthesis suggests that multicomponent^48^ and continuous education involving multidisciplinary teams^49^ rather than once-off interventions would help promote respectful care.

### Limitations and strengths

There are some limitations to our study. Women with perinatal deaths, neonatal deaths or early pregnancy loss would not have been included due to the data collection occurring at neonatal units and postnatal clinics. These women may report different experiences to women who had a live birth.^50^ Women were interviewed at a mean time of 7 weeks post partum and the data are self-reported, therefore, could be subject to recall and social desirability bias. To reduce the likelihood of bias almost all women were recruited at a postnatal clinic which was not the facility where they gave birth. Only a subsample (less than 10%) of women with small and sick newborns were interviewed at the neonatal unit in the hospital where they gave birth. We were unable to determine the indication for caesarean delivery (emergency or elective). The experiences of care could differ between these two indications. These findings are only representative of the experiences of women receiving care in these rural district hospitals. Experiences in larger urban hospitals with obstetric specialists and in private hospitals, where caesarean delivery rates are 74%,^51^ may differ. It is possible that some women who did not attend clinics for postnatal care may have been missed but this is likely to be few as coverage of immunisations for infants under 1 year was 99% for uMzinyathi and 93% for Zululand in 2020.^30^

A strength of our study is that we used a standard validated tool (validated outside SA) with a large sample of women and the largest sample to measure experiences of maternity care among women who had a caesarean delivery. The questions are comprehensive, going beyond mistreatment or disrespect and abuse, to capture the three domains of experience of care from the WHO vision for quality of maternal and newborn health.^52^ The full scale and subscales have good internal consistency reliability in our sample, with a Cronbach’s α value of over 0.8 for the full scale and ranging between 0.63 and 0.79 for the subscales.

There are both strengths and limitations to the use of a scale to measure PCMC. While this scale includes a combination of interpersonal interactions, standards and health system constraints, aggregation of the items into a single scale score can be difficult to interpret. The total scale and subscale results could be used to monitor changes over time within one setting and to compare different settings that have applied the same tool but it may also be difficult for local programme planners and managers to determine what level on the scale would be deemed an acceptable quality of care because recipients of care have different needs and perceptions of what is good care. Identification of tracer indicators that are mandatory in local maternity care policy, such as the offer of a birth companion in SA, may be useful ways of monitoring whether person-centred care has reached an acceptable level.

## Conclusion

SA has made great strides in revising policies and guidelines to prioritise RMC yet this research found that women attending rural health facilities experienced disrespect, lack of support and trust in an environment where they had little involvement in decisions about their care and felt unable to ask questions of their providers. These findings support the need for interventions^48^ addressing infrastructural deficiencies and organisational cultures that allow disrespect within maternity units. In addition, particular aspects of care that require attention include fostering trust with providers and respectful communication, particularly for women having a vaginal delivery, as well as openness to companionship in labour and delivery. Importantly, interventions to improve respectful care should include maternity staff working night shifts.

## supplementary material

10.1136/bmjph-2024-001086 online supplemental table 1

## Data Availability

Data are available on reasonable request.
