# Supplementary material for: Case for improving respectful care: results from a cross-sectional survey of person-centred maternity care in rural South Africa
Source: BMJ Public Health. 2024 Aug 24;2(2):e001086. doi: 10.1136/bmjph-2024-001086 (PMC11816963; doi:10.1136/bmjph-2024-001086)
Supplement: online supplemental table 1 [file bmjph-2-2-s001.pdf]

Supplementary table 1: Distribution of person-centred maternity care variables by mode of delivery

**Items under dignity and respect subscale**<br>(The dignity and respect subscale has 6 items and scores range from 0 to 18)		
	Vaginal delivery (n = 662)<br>n (%)	Caesarean delivery (n = 246)<br>n (%)
Did the doctors, nurses, or other staff at the facility where you gave birth to your baby treat you with respect?		
0 No, never	22 (3)	5 (2)
1 Yes, a few times	29 (5)	5 (2)
2 Yes, most of the time	154 (23)	63 (26)
3 Yes, all of the time	457 (69)	173 (70)
Did the doctors, nurses or other health care providers treat you in a friendly manner?		
0 No, never	52 (8)	9 (4)
1 Yes, a few times	37 (6)	15 (6)
2 Yes, most of the time	147 (22)	68 (28)
3 Yes, all of the time	426 (64)	154 (62)
Did you feel the doctors, nurses or other health care providers shouted at you, scolded, insulted, threatened, or talked to you rudely?		
0 No, never	520 (78)	198 (80)
1 Yes, once	46 (7)	16 (7)
2 Yes, a few times	52 (8)	21 (9)
3 Yes, many times	44 (7)	11 (4)
During your labour or delivery did you feel like you were treated roughly like pushed, beaten, slapped, pinched, physically restrained or gagged?		
0 No, never	618 (93)	229 (93)
1 Yes, once	24 (4)	9 (3)
2 Yes, a few times	9 (1)	4 (2)
3 Yes, many times	11 (2)	4 (2)
During examinations in the labour room, were you given privacy?		
0 No, never	136 (20)	32 (13)
1 Yes, a few times	18 (2)	7 (3)
2 Yes, most of the time	93 (14)	43 (17)
3 Yes, all of the time	414 (64)	163 (66)
4 = Not applicable	1 (0.1)	1 (0.4)
Do you feel like your health information was or will be kept confidential at the facility where you gave birth?		
0 No, never	70 (11)	17 (7)
1 Yes, a few times	35 (5)	17 (7)
2 Yes, most of the time	203 (31)	80 (32)
3 Yes, all of the time	354 (53)	132 (54)
**Items under communication and autonomy subscale**<br>The communication and autonomy subscale has 9 items and scores range from 0 to 27.		
	Vaginal delivery (n = 662)	Caesarean delivery (n = 246)
During your time in the health facility where you gave birth did the doctors, nurses, or other health care providers introduce themselves to you when they first came to see you?		
0 No, none of them	364 (55)	62 (25)

1 Yes, a few of them	120 (18)	66 (27)
2 Yes, most of them	75 (11)	56 (23)
3 Yes, all of them	103 (16)	62 (25)
Did the doctors, nurses, or other health care providers call you by your name/surname (Acceptable name)?		
0 No, never	50 (7)	10 (4)
1 Yes, a few times	39 (6)	5 (2)
2 Yes, most of the time	163 (25)	67 (27)
3 Yes, all the time	410 (62)	164 (67)
Did you feel like the doctors, nurses or other staff at the facility where you gave birth involved you in decisions about your care?		
0 No, never	178 (27)	53 (21)
1 Yes, sometimes	35 (5)	14 (6)
2 Yes, most of the time	99 (15)	44 (18)
3 Yes, all the time	240 (36)	125 (51)
4 Did not have to make any decisions	110 (17)	10 (4)
During the delivery, do you feel like you were able to be in the position of your choice?		
0 No, never	566 (86)	120 (49)
1 Yes, a few times	7 (1)	1 (0.4)
2 Yes, most of the time	28 (4)	104 (42)
3 Yes, all the time	61 (9)	21 (8)
Did the doctors, nurses, or other staff at the facility where you gave birth speak to you in a language you could understand?		
0 No, never	9 (1)	2 (1)
1 Yes, a few times	6 (1)	4 (2)
2 Yes, most of the time	132 (20)	50 (20)
3 Yes, all the time	515 (78)	190 (77)
Did the doctors, nurses, or other staff at the facility where you gave birth ask your permission or consent before doing examinations or procedures on you?		
0 No, never	305(46)	83 (34)
1 Yes, a few times	45 (7)	17 (7)
2 Yes, most of the time	109 (16)	65 (26)
3 Yes, all the time	203 (31)	81 (33)
Did the doctors and nurses explain to you why they were doing examinations or procedures on you?		
0 No, never	247 (37)	71 (29)
1 Yes, a few times	66 (10)	28 (11)
2 Yes, most of the time	123 (19)	58 (24)
3 Yes, all the time	226 (34)	89 (36)
Did the doctors and nurses explain to you why they were giving you any medicine?		
0 No, never	192 (29)	61 (25)
1 Yes, a few times	64 (10)	26 (11)
2 Yes, most of the time	54 (8)	28 (11)
3 Yes, all the time	159 (24)	96 (39)
4 Did not get any medicine	193 (29)	35 (14)

Did you feel you could ask the doctors, nurses or other staff at the facility where you gave birth any questions you had?		
0 No, never	312 (47)	83 (34)
1 Yes, a few times	76 (11)	43 (17)
2 Yes, most of the time	90 (14)	58 (24)
3 Yes, all the time	184 (28)	62 (25)
**Items under supportive care subscale** The supportive-care subscale has 15 items and scores range from 0 to 45.		
How did you feel about the amount of time you waited from the time you arrived in the hospital to the time you were admitted to labour ward?		
0 Very short	258 (39)	83 (34)
1 Somewhat short	190 (29)	59 (24)
2 Somewhat long	104 (16)	50 (20)
3 Very long	110 (16)	54 (22)
Did the doctors and nurses at the facility where you gave birth talk to you about how you were feeling?		
0 No, never	209 (32)	65 (26)
1 Yes, a few times	83 (12)	31 (13)
2 Yes, most of the time	151 (23)	74 (30)
3 Yes, all the time	219 (33)	76 (31)
Did the doctors, nurses, or other staff at the facility where you gave birth try to understand your anxieties/concerns?		
0 No, never	199 (30)	57 (23)
1 Yes, a few times	118 (18)	41 (17)
2 Yes, most of the time	166 (25)	78 (32)
3 Yes, all the time	179 (27)	70 (28)
When you needed help, did you feel the doctors, nurses, or other staff at the facility paid attention?		
0 No, never	106 (16)	23 (9)
1 Yes, a few times	94 (14)	33 (13)
2 Yes, most of the time	207 (31)	95 (39)
3 Yes, all the time	255 (39)	95 (39)
Do you feel the doctors or nurses did everything they could to help control your pain?		
0 No, never	184 (28)	24 (10)
1 Yes, a few times	86 (13)	28 (11)
2 Yes, most of the time	154 (23)	83 (34)
3 Yes, all the time	238 (36)	111 (45)
Were you allowed to have someone you wanted (outside of staff at the facility, such as family or friends) to stay with you during labour?		
0 No, never	582 (88)	210 (85)
1 Yes, a few times	3 (0.4)	2 (1)
2 Yes, most of the time	9 (1)	8 (3)
3 Yes, all the time	21 (3)	10 (4)
4 I did not want someone to stay with me	47 (7)	16 (7)
Were you allowed to have someone you wanted to stay with you during delivery?		
0 No, never	588 (88)	214 (87)
1 Yes, a few times	1 (0.2)	0

2 Yes, most of the time	8 (1)	3 (1)
3 Yes, all the time	12 (2)	8 (3)
4 I did not want someone to stay with me	53 (8)	21 (9%)
Did you feel the doctors, nurses, or other staff at the facility where you gave birth took the best care of you?		
0 No, never	50 (8)	12 (5)
1 Yes, a few times	116 (17)	34 (14)
2 Yes, most of the time	236 (36)	113 (46)
3 Yes, all the time	260 (39)	87 (35)
Did you feel you could completely trust the doctors, nurses, or other staff at the facility where you gave birth with regards to your care?		
0 No, never	68 (10)	16 (6)
1 Yes, a few times	106 (16)	40 (16)
2 Yes, most of the time	189 (29)	81 (33)
3 Yes, all the time	299 (45)	109 (44)
Do you think there were enough health staff in the facility where you gave birth to care for you?		
0 No, never	36 (5)	7 (3)
1 Yes, a few times	113 (17)	41 (17)
2 Yes, most of the time	192 (29)	91 (37)
3 Yes, all the time	321 (49)	107 (43)
Thinking about the labour and postnatal wards, did you feel the health facility was crowded?		
0 No, never	298 (45)	86 (35)
1 Yes, a few times	53 (8)	19 (8)
2 Yes, most of the time	100 (15)	49 (20)
3 Yes, all the time	211 (32)	92 (37)
Thinking about the wards, bathrooms, and the general environment of the health facility where you gave birth, would you say the facility was very clean, clean, dirty or very dirty?		
0 Very dirty	59 (9)	21 (8)
1 Dirty	66 (10)	26 (11)
2 Clean	458 (69)	170 (69)
3 Very clean	79 (12)	29 (12)
Was there water at the facility where you gave birth?		
0 No, never	69 (10)	14 (6)
1 Yes, a few times	159 (24)	69 (28)
2 Yes, most of the time	143 (22)	69 (28)
3 Yes, all the time	291 (44)	94 (38)
Was there electricity at the facility where you gave birth?		
0 No, never	6 (1)	3 (1)
1 Yes, a few times	158 (24)	69 (28)
2 Yes, most of the time	109 (16)	44 (18)
3 Yes, all the time	389 (59)	130 (53)
In general, did you feel safe in the health facility where you gave birth?		
0 No, never	23 (4)	8 (3)
1 Yes, a few times	22 (3)	8 (3)
2 Yes, most of the time	161 (24)	70 (29)
3 Yes, all the time	456 (69)	160 (65)

Additional questions not included in the PCMC scale		
At any time during your stay in the health facility, did you share a bed with another patient		
0 No, never	586 (89)	220 (90)
1 Yes, some of the time	50 (7)	18 (7)
2 Yes, all of the time	26 (4)	8 (3)
If you had a choice, how likely is it that you would choose to give birth in that facility again?		
1 Definitely not	83 (12)	37 (15)
2 Probably not	43 (7)	9 (4)
3 Maybe	74 (11)	31 (12)
4 Definitely yes	461 (70)	169 (69)
